# Dramatic Increases of Soil Microbial Functional Gene Diversity at the Treeline Ecotone of Changbai Mountain

**DOI:** 10.3389/fmicb.2016.01184

**Published:** 2016-07-29

**Authors:** Congcong Shen, Yu Shi, Yingying Ni, Ye Deng, Joy D. Van Nostrand, Zhili He, Jizhong Zhou, Haiyan Chu

**Affiliations:** ^1^State Key Laboratory of Soil and Sustainable Agriculture, Institute of Soil Science, Chinese Academy of SciencesNanjing, China; ^2^State Key Laboratory of Urban and Regional Ecology, Research Center for Eco-Environmental Sciences, Chinese Academy of SciencesBeijing, China; ^3^University of the Chinese Academy of SciencesBeijing, China; ^4^CAS Key Laboratory of Environmental Biotechnology, Research Center for Eco-Environmental Sciences, Chinese Academy of SciencesBeijing, China; ^5^Institute for Environmental Genomics and Department of Microbiology and Plant Biology, University of Oklahoma, NormanOK, USA; ^6^State Key Laboratory of Environment Simulation and Pollution Control, School of Environment, Tsinghua UniversityBeijing, China; ^7^Earth and Environmental Sciences, Lawrence Berkeley National Laboratory, BerkeleyCA, USA

**Keywords:** metagenomics, GeoChip, microbial functional genes, bacterial taxonomic and phylogenetic diversity, alpha and beta diversity patterns, treeline ecotone, elevation gradient, soil dissolved organic carbon

## Abstract

The elevational and latitudinal diversity patterns of microbial taxa have attracted great attention in the past decade. Recently, the distribution of functional attributes has been in the spotlight. Here, we report a study profiling soil microbial communities along an elevation gradient (500–2200 m) on Changbai Mountain. Using a comprehensive functional gene microarray (GeoChip 5.0), we found that microbial functional gene richness exhibited a dramatic increase at the treeline ecotone, but the bacterial taxonomic and phylogenetic diversity based on 16S rRNA gene sequencing did not exhibit such a similar trend. However, the β-diversity (compositional dissimilarity among sites) pattern for both bacterial taxa and functional genes was similar, showing significant elevational distance-decay patterns which presented increased dissimilarity with elevation. The bacterial taxonomic diversity/structure was strongly influenced by soil pH, while the functional gene diversity/structure was significantly correlated with soil dissolved organic carbon (DOC). This finding highlights that soil DOC may be a good predictor in determining the elevational distribution of microbial functional genes. The finding of significant shifts in functional gene diversity at the treeline ecotone could also provide valuable information for predicting the responses of microbial functions to climate change.

## Introduction

With the rapid development of molecular tools, great progress on microbial biogeography has been made over the past decade (Martiny et al., 2006; Green et al., 2008; Hanson et al., 2012; Zhou et al., 2016). Researchers examining biogeographic patterns are now focusing on functional traits rather than just taxa (Fierer et al., 2012; Hanson et al., 2012; Fierer et al., 2013). Patterns of functional traits provide a better understanding of ecosystem functions, biogeochemical cycles and microbial responses to environmental change, which can shed light on fundamental questions in biology (Petchey and Gaston, 2002; Green et al., 2008; Fuhrman, 2009; Reiss et al., 2009). Recent studies have focused on functional gene distribution in soil microbial communities across large-scale natural ecosystems or latitudinal gradients (Fierer et al., 2012, 2013; Shi et al., 2015). There were also studies about the functional gene distribution along elevation, but the range and scale of elevational gradients were relatively small (Yang et al., 2014; Ding et al., 2015).

The high altitude limit of forests, commonly referred to as the treeline, timberline or forest line, has obtained extensive attention due to its sensitivity to climate change (Körner and Paulsen, 2004; Holtmeier and Broll, 2007). In reality, the transition from the uppermost closed montane forests to the treeless alpine vegetation is not really a line, but rather a steep gradient of increasing tree stands fragmentation and stunted growth, often called the treeline ecotone (Körner and Paulsen, 2004). However, little research has focused on the soil microbial communities at the treeline in elevational studies, until recently when Thébault et al. (2014) examined these communities with phospholipid fatty acid analysis (PLFA) technique and observed close correlations between microbial taxonomic communities and nutrient availability. Ding et al. (2015) examined the functional gene structure at the forest timberline using GeoChip technology, and they found that functional gene compositions were significantly different between shrubland and coniferous forest. Nonetheless, we do not know if microbial elevational diversity patterns will change in treeline ecotone and if microbial taxa and functional genes show the same pattern along an elevation gradient.

The Changbai Mountain is the highest mountain in Northeast China and is one of the most well-protected and conserved natural ecosystems on Earth (He et al., 2005). The elevational distribution of vegetation along the mountainside mirrors the latitudinal vegetation gradient from temperate to frigid zones on the Eurasian continent (Xu et al., 2004; Zhang et al., 2011). The treeline ecotone is at 1900–2000 m at the transition from birch forest to tundra. In this study, we hypothesized that soil microbial community would show different elevational diversity patterns due to different environmental controls. We used GeoChip 5.0, a functional gene microarray, and 16S rRNA gene sequencing to examine microbial functional gene and taxonomic diversity along an elevation gradient on Changbai Mountain. We found that microbial functional gene richness exhibited a dramatic increase at the treeline ecotone, but the bacterial taxonomic and phylogenetic diversity did not exhibit such a similar trend. Our results indicated that the elevational α-diversity patterns for bacterial taxa and functional genes were quite different, but that β-diversity patterns were similar. Our results also indicated that soil DOC might be a good predictor of microbial functional gene elevational distribution. This work provides valuable information for predicting the response of microbial functions to climate change.

## Materials and Methods

### Site Selection and Soil Sampling

As the highest mountain in north-eastern China and the head of three large rivers (the Songhua, Yalu and Tumen), Changbai Mountain (126°55′–129°00′E; 41°23′–42°36′N) is located in Jilin Province, which extends along the border of China and North Korea. Topographic and climatic variations result in a distinct vertical zonation of major forest types, especially along the northern slope. It has a typical continental temperate monsoon climate. Along the elevational gradient from 530 to 2200 m, the mean annual temperature decreases from 2.9 to -4.8°C, and the mean annual precipitation increases from 632 to 1154 mm (Tong et al., 2010). Below 1100 m there is a typical temperate forest, mainly composed of Korean pine and hardwood species; from 1100 to 1700 m, evergreen coniferous forest, dominated by spruce and fir, is typical; from 1700 to 2000 m is mountain birch forest; and above 2000 m is unique alpine tundra, which marks the southernmost occurrence of this ecosystem type on the eastern Eurasian continent. The forest-tundra treeline ecotone is present from 1900 to 2000 m (Supplementary Figure S1). The main climatic and ecological characteristics along the elevational gradient are summarized in Supplementary Table S1.

We collected soil samples from the northern slope of Changbai Mountain on 30 August 2013 at seven elevations (500, 700, 1000, 1300, 1600, 1900, and 2200 m). At each elevation, soil samples were collected from 5 plots (10 m × 10 m) as five independent replicates. In each plot, samples of the soil organic layer (∼10 cm × 10 cm in area, and 0–5 cm depth directly below the litter layer) were collected at ten random points using a sterile blade and composited together as a single sample. Visible roots and residue were removed from each sample prior to homogenizing the soil fraction. The fresh soil samples were sieved through 2-mm meshes and subdivided into two subsamples. One was stored at 4°C to determine the physical and chemical properties, and the other was stored at -20°C for subsequent DNA extraction.

### Soil Physicochemical Analyses

Soil pH was measured using a pH meter (FE20-FiveEasy^TM^ pH, Mettler Toledo, Germany) after shaking a soil water (1:5 w/v) suspension in the shaker for 30 min. Soil moisture was measured gravimetrically. Total carbon (TC) and total nitrogen (TN) contents were measured by elemental analyzer (Vario MAX, Elementar, Germany). Ammonium (NH_4_^+^-N), nitrate (NO_3_^-^-N), dissolved organic carbon (DOC) and dissolved total nitrogen (DTN) were extracted at a ratio of 10 g fresh soil to 100 mL 2 M KCl. After shaking for 1 h, ${\mathrm{NH}}_{4}^{+}$-N, ${\mathrm{NO}}_{3}^{-}$-N and DTN in the extracts (filtering through 10 cm diameter quantitative filter paper) were analyzed using a continuous flow analytical system (San^++^ System, Skalar, Holland), and DOC was determined using a TOC analyzer (Multi N/C 3000, Analytik Jena, Germany). Dissolved organic nitrogen (DON) was calculated as follows: DON = DTN -${\mathrm{NH}}_{4}^{+}$-N -${\mathrm{NO}}_{3}^{-}$-N. Available P (AP) in soil was extracted by sodium bicarbonate and determined using the molybdenum blue method (Olsen et al., 1954). Available K (AK) in soil was extracted by ammonium acetate and determined by flame photometry (Carson, 1980).

### DNA Extraction

Soil DNA was extracted using a PowerSoil DNA kit (MO BIO, Carlsbad, CA, USA), and then purified with an UltraClean 15 DNA purification kit (MO BIO, Carlsbad, CA, USA). DNA concentrations were estimated by electrophoresis on 1% agarose gels, and DNA was diluted to 20 ng/μl before use in PCR.

### Illumina MiSeq Sequencing and Data Processing

16S rRNA genes were amplified using a common primer set (515F, 5′-GTGCCAGCMGCCGCGGTAA-3′; 806R, 5′-GGACTACHVGGGTWTCTAAT-3′) combined with adapter sequences and barcode sequences (Caporaso et al., 2011). PCR amplifications were conducted with 25 μl 2× premix (TaKaRa, Otsu, Japan), 0.5 μl 20 mM each forward and reverse primer, and 50 ng of DNA, and the volume was completed to 50 μl with double-distilled water. Each sample was amplified in triplicate using 30 cycles (94°C for 30 s, 55°C for 30 s, and 72°C for 30 s) with a final extension at 72°C for 10 min. The three reaction products were pooled and purified using a QIAquick PCR purification kit (QIAGEN, Shenzhen, China) and then quantified using a NanoDrop ND-1000 spectrophotometer (Thermo Scientific, Wilmington, NC, USA). PCR products were sequenced on two lanes of a 2 × 151 bp sequencing run on an Illumina MiSeq (Caporaso et al., 2012).

Raw data were processed and analyzed as previously described (Caporaso et al., 2012), using the QIIME software and following the workflow^1^. Briefly, sequences were quality filtered (max value of 0.5) and clustered into 97% similar phylotypes after removing singleton sequences. Taxonomy was identified using the Ribosomal Database Project classifier (Wang et al., 2007) which was trained on the Greengenes 13_8 16S rRNA database (McDonald et al., 2012). To rarify all the data sets to the same level of sampling effort, 4000 sequences were randomly selected. Sequences were deposited to the MG-RAST metagenomics analysis server^2^ and are available to the public (accession numbers from 4706639.3 to 4706673.3).

### GeoChip Hybridization and Data Processing

DNA was extracted using a PowerSoil DNA kit as described above. GeoChip 5.0 was used to analyze DNA samples as described previously (Wang et al., 2014). Briefly, DNA (1 μg) was labeled with the fluorescent dye Cy-3 using a random priming method and then purified with the QIA quick purification kit (QIAGEN, Shenzhen, China) according to the manufacturer’s instructions. After purification, the labeled DNAs were hybridized with the Agilent platform-based GeoChip 5.0 arrays at 67°C plus 10% formamide for 24 h. GeoChip microarrays were scanned with a 100% laser power and 100% photomultiplier tube with a NimbleGen MS 200 Microarray Scanner (Roche, Basel, Switzerland). Spots with a signal-to-noise ratio <2.0, signals <150, or <1.3 times the background were discarded prior to statistical analyses. Raw data were quantified and processed using the analysis pipeline as previously described (He et al., 2010; Tu et al., 2014). Then processed GeoChip data were analyzed using the following steps: (i) removing genes detected in less than 3 of 5 samples from the same elevation; (ii) normalizing the signal intensity of each spot by dividing the signal intensity by the total intensity of the microarray followed by multiplying by a constant; and (iii) transforming data to the natural logarithm.

### Statistical Analysis

Bray–Curtis similarity distance based on bacterial OTU table and functional gene table was used to calculate bacterial dissimilarities and microbial functional gene dissimilarities. Principal co-ordinates analysis (PCoA) were performed on the basis of Bray–Curtis dissimilarities. The variations in beta diversity were plotted against changes in elevational distance to discern any patterns. The distance–decay relationship (which measures how similarity decays with increasing distance between pairwise sites) was analyzed using a Gaussian generalized linear model, and the significance was determined using Pearson’s correlation. In order to identify environmental and biogeochemical factors associated with functional gene diversity, correlations between diversity metrics and GeoChip data were conducted by SPSS software (version 20.0). Mantel tests of Bray-Curtis similarity distance values were calculated and carried out within vegan package in R (R Development Core Team, 2010; Dixon, 2003). The multivariate regression tree was created within the mvpart package in R (De’Ath, 2002). Distance-based linear model multivariate analysis (DistLM) (McArdle and Anderson, 2001) was implemented to determine the influence of environmental variables on microbial functional gene composition. Marginal tests were applied to assess the statistical significance and percentage contribution of each variable in isolation, and sequential tests were performed to evaluate the cumulative effect of environmental variables.

## Results

### General Characteristics of Soil Microbial Communities

We obtained 329,598 high quality sequences from 16S rDNA amplicon sequencing for all soil samples (4,321 to 20,159 sequences per sample). A total of 30,302 unique OTUs were identified and were assigned to 43 bacterial phyla. Among the identified phyla, *Proteobacteria* (23.1%), *Acidobacteria* (20.8%), and *Verrucomicrobia* (17.29%) were the most abundant phyla across the seven elevations (Supplementary Table S4). Other dominant phyla across all the Changbai Mountain soils were Actinobacteria (5.92%), Bacteroidetes (5.76%), Chloroflexi (4.27), Planctomycetes (4.47%), Firmicutes (7.21%), accounting for more than 27% of the bacterial sequences from each of the soils (Supplementary Figure S3). In addition, *Nitrospirae, Gemmatimonadetes, Armatimonadetes* and other 29 phyla were present in most soils but at relatively low abundances (Supplementary Table S4).

**FIGURE 1:**
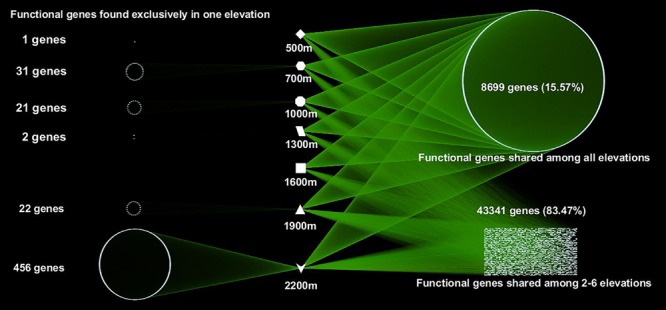
**A network map showing the interactions of microbial functional genes among all the samples from different elevations.** Each point represents one independent functional gene. Functional genes in the left column were unique to one site, while those in the right belonged to multiple elevation sites.

**FIGURE 2:**
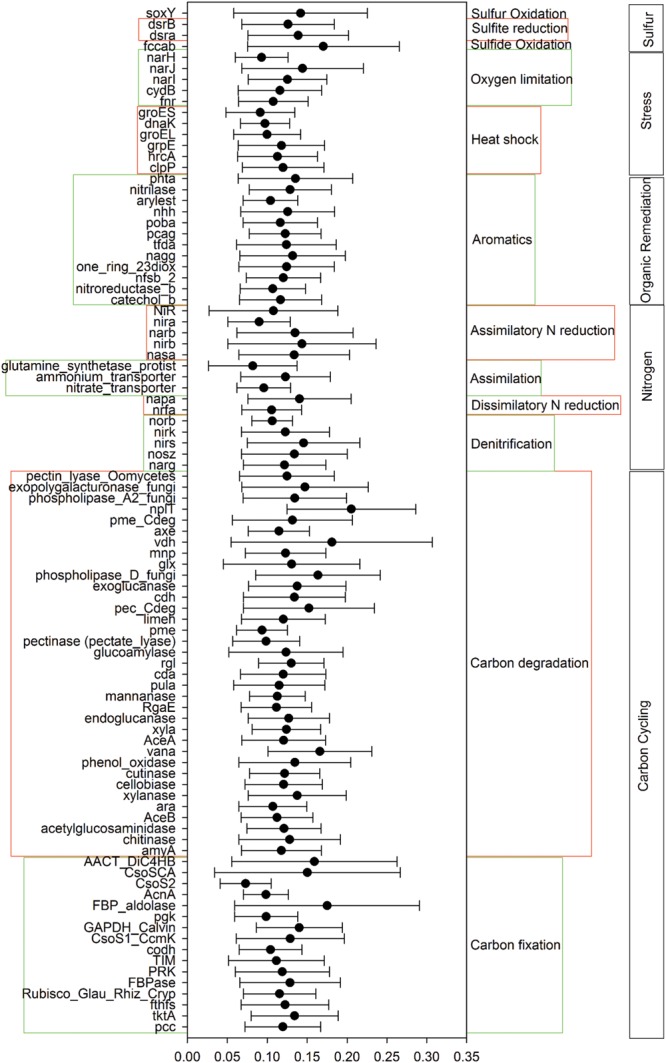
**Specific functional gene categories that exhibited significant changes in abundance from coniferous forest (1600 m) to bich forest at the treeline ecotone (1900 m).** Significance was determined using the response ratio analysis at a 95% confidence interval (CI).

A total of 55,874 functional genes in 35 samples were detected by GeoChip 5.0. These genes were mainly involved in six functional categories: carbon (C)-cycling (15.9%), nitrogen (N)-cycling (4.2%), environmental stress response (15.3%), metal homeostasis (26.2%), organic remediation (8.9%) and virulence (16.2%). Other functional categories involved in sulfur and phosphorus cycling, and electron transfer accounted for a small proportion (Supplementary Table S2). Functional genes shared among all elevations accounted for 15.57% of the detected genes, while those shared among 2–6 elevations accounted for 83.47%. The greatest number of unique functional genes (functional genes found exclusively in one elevation) were detected at 2200 m (456 functional genes), suggesting that the tundra ecosystem has more unique functional genes than forest ecosystems (**Figure 1**). Specific genes involved in carbon and nitrogen cycling like *nifH, nosZ, ureC*, CODH genes, *mcrA*, and *pmoA*, all show a higher gene richness at 1900 and 2200 m (Supplementary Table S3). Response ratio analysis showed that specific functional gene categories that exhibited significant (95% CI) higher gene abundances from coniferous forest to birch forest at the treeline ecotone are mainly involved in carbon degradation and fixation (**Figure 2**).

### Alpha and Beta Diversity Patterns

**FIGURE 3:**
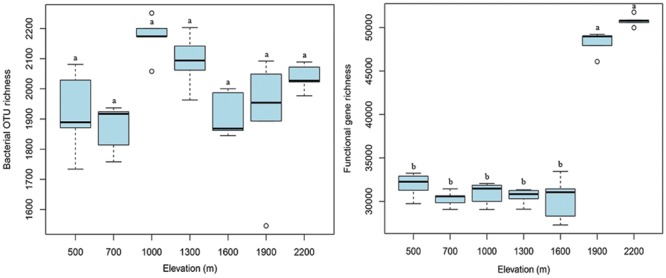
**The elevational alpha diversity patterns for bacterial communities and microbial functional genes.** Bacterial alpha diversity (OTU richness) exhibited no apparent elevational gradient, whereas microbial functional gene alpha diversity (functional gene richness) showed a sharp increase at 1900 m (treeline ecotone). Different letters indicate significant differences (*p* < 0.05).

At the taxonomic and phylogenetic level, the alpha diversity of bacterial communities (randomly selected 4,000 sequences per sample) did not show an apparent trend along the elevation gradient (**Figure 3**), which is consistent with our previous results (454 data in Shen et al., 2013). The number of functional genes detected was significantly (*p* < 0.05) higher at 1900 and 2200 m than at lower elevations, resulting in a sharp increase in functional gene richness at the treeline ecotone (**Figure 3**). These results suggest that the treeline ecotone has a strong influence on microbial functional gene richness, rather than bacterial diversity.

**FIGURE 4:**
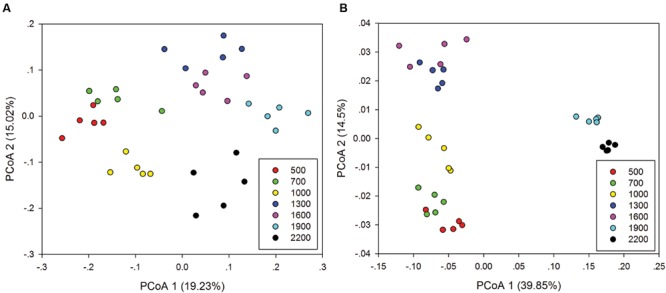
**Principal co-ordinates analysis (PCoA) of the bacterial community composition **(A)** and microbial functional gene composition **(B)****.

**FIGURE 5:**
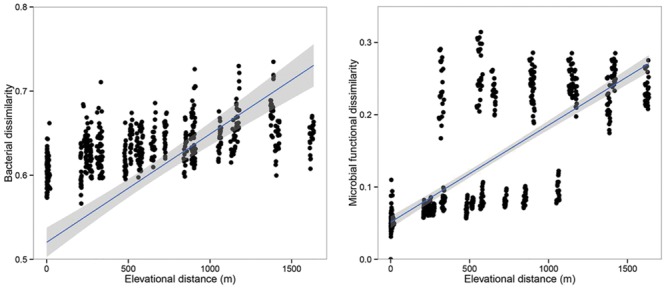
**The relationships between the community dissimilarity (bacterial communities and microbial functional genes) and elevational change.** Both the bacterial communities and microbial functional genes showed significant elevational distance-decay patterns (*r* = 0.409, *p* < 0.001; *r* = 0.708, *p* < 0.001).

Principal co-ordinates analysis analysis based on 16S rRNA gene sequencing and GeoChip datasets showed that both bacterial and functional gene composition tended to be relatively similar among samples within the same elevation and distinctly different among the different elevations (**Figure 4**). Dissimilarity tests revealed that bacterial composition and functional gene composition differed significantly (*p* < 0.05) among elevations (Supplementary Table S6; **Table 1**). Furthermore, both the bacterial (*r* = 0.409, *P* < 0.001) and microbial functional gene (*r* = 0.708, *P* < 0.001) compositional dissimilarities significantly increased with increasing elevations, which showed significant elevational distance-decay patterns (**Figure 5**). It is noteworthy that functional gene data separated into two groups based on which side of the treeline ecotone the samples were collected (**Figure 5**). This suggests that effect of treeline ecotone on functional gene composition is bigger than that on bacterial composition. Nevertheless, these results suggest that the elevational alpha diversity patterns for bacterial taxa and functional genes are different, but they could exhibit similar elevational beta diversity patterns.

**Table 1:** Results from analysis of microbial functional gene composition dissimilarities (MRPP, ADONIS, and ANOSIM) among different elevations.

	MRPP		ADONIS		ANOSIM	
	δ	*p*	*F*	*p*	*R*	*p*
500 vs. 700	0.05	0.005	4.56	0.001	0.828	0.007
500 vs. 1000	0.052	0.01	4.338	0.001	0.752	0.006
500 vs. 1300	0.052	0.008	7.462	0.002	1	0.004
500 vs. 1600	0.063	0.01	5.654	0.001	0.732	0.007
500 vs. 1900	0.05	0.013	83.661	0.005	1	0.007
500 vs. 2200	0.048	0.008	108.418	0.005	1	0.007
700 vs. 1000	0.051	0.012	4.758	0.001	0.928	0.011
700 vs. 1300	0.051	0.007	6.887	0.002	1	0.01
700 vs. 1600	0.062	0.008	4.288	0.001	0.648	0.007
700 vs. 1900	0.049	0.005	107.31	0.003	1	0.006
700 vs. 2200	0.048	0.005	137.503	0.001	1	0.01
1000 vs. 1300	0.053	0.006	4.485	0.001	0.892	0.009
1000 vs. 1600	0.065	0.01	3.445	0.001	0.608	0.004
1000 vs. 1900	0.051	0.01	89.406	0.01	1	0.007
1000 vs. 2200	0.05	0.009	114.763	0.003	1	0.007
1300 vs. 1600	0.065	0.006	3.261	0.001	0.572	0.005
1300 vs. 1900	0.051	0.013	94.395	0.001	1	0.012
1300 vs. 2200	0.05	0.009	121.4	0.005	1	0.011
1600 vs. 1900	0.062	0.014	61.476	0.001	1	0.011
1600 vs. 2200	0.061	0.011	77.658	0.002	1	0.008
1900 vs. 2200	0.048	0.009	5.843	0.002	1	0.011

### The Key Environmental Factors Influencing the Diversity and Composition of Soil Bacterial Community and Microbial Functional Genes

In terms of OTU richness and Faith’s PD, bacterial OTU richness was significantly (*p* < 0.05) correlated with soil pH, moisture, ${\mathrm{NO}}_{3}^{-}$-N, TC and TN, while bacterial phylogenetic diversity was significantly correlated with soil pH and AK (Supplementary Table S5). Mantel tests showed that pH had the highest correlation with bacterial community composition (*r* = 0.36, *P* < 0.001, **Table 2**). Specifically, soil pH showed a significant correlation with the relative abundance of the four dominant phyla (Supplementary Figure S4).

**Table 2:** Mantel test results for the correlation between bacterial community composition and microbial functional gene composition and environmental variables along the elevational gradient.

	Bacteria		Functional genes	
Variable	*r*	*p*	*r*	*p*
pH	**0.36**	**<0.001**	-0.02	0.58
AP	**0.31**	**<0.001**	0.11	0.08
%TN	**0.27**	**<0.001**	-0.05	0.80
DOC	**0.25**	**0.01**	**0.52**	**<0.001**
DON	**0.22**	**0.02**	**0.4**	**<0.001**
${\mathrm{NO}}_{3}^{-}$-N	**0.22**	**0.02**	-0.02	0.55
${\mathrm{NH}}_{4}^{+}$-N	**0.19**	**0.03**	0.11	0.11
AK	**0.13**	**0.04**	-0.03	0.67
Moisture	**0.12**	**0.05**	0.03	0.28
%TC	0.12	0.07	**0.13**	**0.03**

*Values in bold indicate significant correlation (*p* < 0.05). TC, total carbon; TN, total nitrogen; DOC, dissolved organic carbon; DON, dissolved organic nitrogen; ${\mathrm{NH}}_{4}^{+}$-N, ammonium; ${\mathrm{NO}}_{3}^{-}$-N: nitrate; AP, available P; AK, available K.*

**FIGURE 6:**
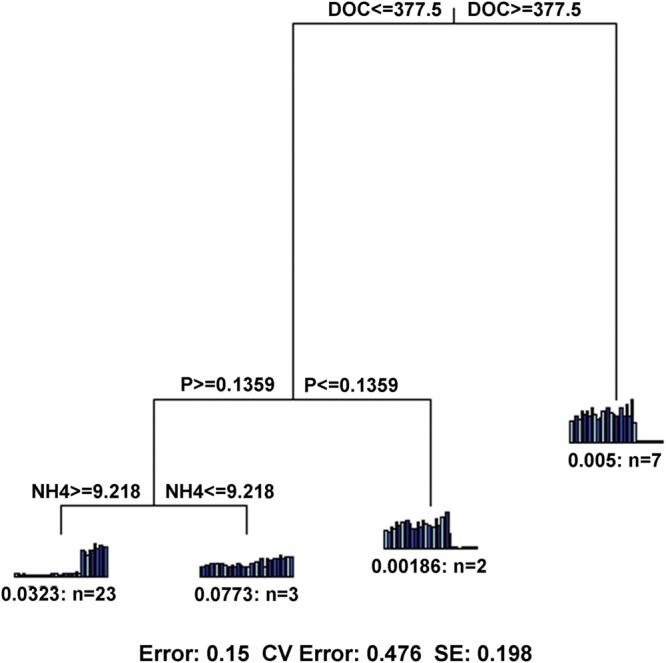
**Multivariate regression tree (MRT) showing the most important factor with microbial functional gene composition.** DOC, dissolved organic carbon; NH4, ammonium; P, available P.

In terms of functional gene richness and Shannon index, soil DOC, DON, TC, moisture, AP and ${\mathrm{NO}}_{3}^{-}$-N showed significant (*p* < 0.05) correlations with functional gene diversity (Supplementary Table S5). Mantel tests showed that the microbial functional gene composition was significantly (*p* < 0.05) correlated with soil DOC, DON and TC (**Table 2**). Of all of the environmental variables examined, DOC showed the highest correlation with the functional structure based on Multivariate Regression Trees (MRT) analyses (**Figure 6**). Sequential tests of DistLM analysis indicated four significant variables (DOC, AP, AK, ${\mathrm{NO}}_{3}^{-}$-N) that explained 62.3% of the total variation of microbial functional gene composition, with soil DOC providing the greatest explanatory power (43.5% of the total variation; **Table 3**). These results suggest that the bacterial community composition and microbial functional gene diversity were influenced by different drivers with the bacterial diversity and community composition being strongly influenced by soil pH, while the microbial functional gene diversity and composition were significantly correlated with soil DOC.

**Table 3:** Results of distance-based multivariate linear model (DistLM) for microbial functional gene composition showing the % variation explained by environmental variables.

Variable	%Var	pseudo-F	*p*	Cum.(%)
**(a) Variables individually**				
DOC	43.52	25.43	0.001	
DON	28.07	12.88	0.002	
AP	21.75	9.17	0.005	
${\mathrm{NO}}_{3}^{-}$-N	15.34	5.98	0.008	
%TC	17.84	7.16	0.01	
Moisture	11.82	4.42	0.027	
AK	7.89	9.17	0.088	
pH	6.34	2.23	0.14	
%TN	3.51	1.19	0.267	
${\mathrm{NH}}_{4}^{+}$-N	0.77	0.25	0.747	
**(b) Variables fitted sequentially**				
DOC	43.52	25.43	0.001	43.52
AP	7.98	5.26	0.023	51.5
AK	6.63	4.91	0.013	58.13
${\mathrm{NO}}_{3}^{-}$-N	4.14	3.28	0.036	62.27
${\mathrm{NH}}_{4}^{+}$-N	3.14	2.64	0.074	65.41
Moisture	3.11	2.75	0.065	68.52
pH	2.07	1.91	0.124	70.59
%TN	1.82	1.71	0.168	72.41
%TC	3.09	3.15	0.058	75.5
DON	0.56	0.55	0.582	76.06

*Var, percentage of variance in species data explained by that variable; Cum. %, cumulative percentage of variance explained. Variable abbreviations as in **Table 2**. TC, total carbon; TN, total nitrogen; DOC, dissolved organic carbon; DON, dissolved organic nitrogen; ${\mathrm{NH}}_{4}^{+}$-N, ammonium; ${\mathrm{NO}}_{3}^{-}$-N, nitrate; AP, available P; AK, available K. (a) each variable analyzed individually (ignoring other variables); (b) forward-selection of variables, where amount explained by each variable added to model is conditional on variables already in the model.*

## Discussion

The elevational diversity gradient is one of the most fundamental patterns in biogeography (Lomolino, 2001; Bryant et al., 2008). There are many lines of evidence showing that microbes do not follow the classic decreasing or unimodal elevational diversity patterns that plants and animals follow. As expected, we observed that soil bacteria exhibited no apparent elevational trend based on taxonomic diversity, which is consistent with previous reports (Fierer et al., 2011; Shen et al., 2013; Xu et al., 2014; Yuan et al., 2014). In contrast, microbial functional gene richness showed a dramatic shift at the treeline ecotone along this elevation gradient. To the best of our knowledge, this finding has not been reported in other elevational studies, suggesting that the primary controls on microbial functional gene diversity may be different from those for taxa. Moreover, microbial functional gene composition significantly differed across elevations, which is in line with a previous study of the Tibetan Plateau (Yang et al., 2014). Given that many researchers have found a clear bacterial or micro-eukaryotic compositional difference among different elevations (Singh et al., 2012; Shen et al., 2014, 2015; Yuan et al., 2014; Jarvis et al., 2015), these results suggest that elevation could be a major driver of variation in both microbial taxonomic and functional gene composition. Beta diversity (compositional dissimilarity among sites) is central to the ecological and evolutionary processes shaping the distribution of species (Condit et al., 2002). Yet elevational patterns of beta diversity have not received as much attention as that of alpha diversity (Wang et al., 2012). Here we found that bacterial community dissimilarities significantly increased with increasing elevation, and that microbial functional genes also exhibited a significant elevational distance-decay pattern, though the treeline ecotone had a bigger influence on functional gene composition than bacterial taxa composition. However, this elevational distance-decay pattern may not be observed in all environments. Bryant et al. (2008) observed that the compositional similarity of soil Acidobacteria significantly decreased with elevational distance, whereas Wang et al. (2012) found that benthic bacteria did not show a significant elevational distance-decay relationship. The elevational distance-decay pattern may be more easily observed in soils than aquatic environments or it could be caused by the greater spatial heterogeneity in soils than that in water. Together, these results refute our hypothesis, and suggest that the elevational alpha diversity patterns for bacterial communities and microbial functional genes are different, although they could have similar elevational beta diversity patterns.

The relationship between taxonomic diversity and functional diversity in soils is largely unknown (Petchey and Gaston, 2002; Rosenfeld, 2002; Allison and Martiny, 2008). Scientists are trying to test this relationship at different scales by combining 16S rRNA gene sequencing with whole genome shotgun sequencing or GeoChip analysis (Fierer et al., 2012, 2013; Ding et al., 2015; Leff et al., 2015; Shi et al., 2015). For example, Fierer et al. (2012) found significant correlations between bacterial diversity and functional gene diversity (include alpha and beta) in different ecosystems across the globe. Furthermore, this correlation seems to become stronger within the single tallgrass prairie ecosystem in the midwestern United States (Fierer et al., 2013). The above-mentioned findings highlight that the overall diversity of functional gene categories found in a given sample is, to some degree, predictable from the taxonomic diversity of the microbial communities. Actually, while the taxonomic and phylogenetic beta diversity is significantly correlated with functional gene beta diversity, the alpha diversity may not show identical manner. For instance, Shi et al. (2015) found no correlation between bacterial richness and microbial functional gene richness across an Arctic tundra ecosystem. Similarly, our study here together with a study in a forest timberline, did not detect this relationship (Ding et al., 2015). The contradictory results could be caused by differences in terms of technique (shotgun metagenome sequencing vs. GeoChip, see Zhou et al., 2015), scale or ecosystem examined. Therefore, more research using multiple comparable techniques are needed to test this relationship (especially for alpha diversity) across different ecosystems. Functional redundancy of species is assumed to be a common feature in soils (Heemsbergen et al., 2004). This concept is based on the observation that some species perform similar roles in communities and ecosystems, and may therefore be substitutable with little impact on ecosystem processes (Lawton and Brown, 1993). Given the non-significant bacterial richness pattern observed along the elevation gradient and the sharp increase of functional gene richness at the treeline ecotone observed in this study, soil bacterial taxa appear to exhibit a high degree of functional redundancy. Of course, functional redundancy cannot be determined simply based on the relationship of taxonomic and functional gene alpha diversity, as redundancy is difficult to establish because it requires detailed knowledge of the microbial populations that perform a specific process (Allison and Martiny, 2008).

Identifying the environmental factors that best explain microbial community variation is a fundamental goal in microbial ecology (Martiny et al., 2006; Hanson et al., 2012). Microbial taxa display spatial patterns linked to geographic distance (Cho and Tiedje, 2000; Green and Bohannan, 2006; Martiny et al., 2006), vegetation type (Knelman et al., 2012; Shen et al., 2013), and soil characteristics (Fierer and Jackson, 2006; Lauber et al., 2009; Chu et al., 2010; Liu et al., 2014). Previous studies have demonstrated that soil pH drives both bacterial horizontal and elevational distribution (Fierer and Jackson, 2006; Chu et al., 2010; Griffiths et al., 2011; Shen et al., 2013). In this study, we found a significant relationship between soil pH and bacterial diversity, which is in line with our previous results based on pyrosequencing (Shen et al., 2013). However, no significant correlations were found here between pH and functional gene diversity and gene composition, suggesting a weak role of soil pH in influencing microbial functional genes.

The sensitivity and response of the treeline to climate change has been increasingly discussed from global to regional and smaller scales (Körner and Paulsen, 2004; Holtmeier and Broll, 2007). In this study, our results showed a dramatic shift of functional gene richness at the treeline ecotone. To our knowledge, this is the first reported observation of a significant shift of functional gene richness from a forest ecosystem to a tundra ecosystem along an elevation gradient. Climate warming leads to movement of the treeline toward a higher elevation, but little is known about the dynamics of microbial functions under this change. Thus, this finding provides a great value in predicting the response of microbial functions to climate change. We concluded this pattern was largely driven by soil DOC, since it was the most significant factor correlated with microbial functional gene diversity. Our study showed that soil DOC content significantly increased with elevation, particularly was higher at the treeline ecotone and tundra (Supplementary Figure S2). Variations in DOC content might caused by differences in vegetation types (see Supplementary Table S1). On one hand, litter input produced by different vegetations can lead to difference in soil organic matter composition (Quideau et al., 2001). On the other hand, vegetation type greatly influences soil physicochemical properties, which forms different soil microclimate (Knelman et al., 2012). DOC is a complex and heterogeneous mixture of C compounds, including humic acids, fulvic acids, hydrophobic neutrals, and hydrophilic compounds, which may be both a substrate for microbial activity and a byproduct of the subsequent microbial metabolic processes, thus providing strong evidence of links between microbial activity and DOC concentrations (Marschner and Kalbitz, 2003; van Hees et al., 2005; Bolan et al., 2011). For instance, previous studies on alpine ecosystems showed that tundra soils at higher elevations had more labile carbon and greater microbial activity than forest soils (Neff and Hooper, 2002; Sjögersten et al., 2003). Using the Biolog method, Tian et al. (2015) found that labile DOC accounted for the largest amount of variation in microbial metabolic functional diversity. Importantly, Straathof (2015) studied the relationship between composition of DOC and microbial respiration, and concluded that the quality of DOC, via regulation of microbial processes, might be an important indicator of soil functions. However, while above-mentioned studies mainly focused on microbial respiration and substrate utilization, we first found significant correlations between functional gene diversity and DOC. Meanwhile, specific functional gene categories that exhibited significant higher gene abundances at the treeline ecotone are mainly involved in carbon degradation and fixation. This suggests that these functional genes associated with C cycling are closely linked with soil C dynamics, which may result in significant differences in microbial metabolic potential between the two sides of treeline. In addition, it should be noted that other factors which were not detected in our study may also have fundamental contributions for microbial functional gene variations. For example, Ding et al. (2015) found soil temperature was the best predictor for microbial functional gene variations. At the same time, the authors noted that the contribution of soil DOC and other nutrient contents to the functional gene variations cannot be overlooked, since temperature is likely an indirect factor affecting the microbial community by controlling soil nutrient availability (Mitchell et al., 2012). Taken together, our results suggest that soil DOC could be a good predictor for microbial functional gene diversity and composition, and this finding provides strong evidence for DOC as an important indicator of soil functions at the functional gene level.

## Conclusion

In summary, we observed the distribution of soil microbial functional gene along a complete elevational gradient on Changbai Mountain. Soil microbial functional gene richness exhibited a dramatic increase at the treeline ecotone, but bacterial diversity did not exhibit a similar elevational trend. Both bacterial taxa and functional genes showed significant elevational distance-decay patterns as their dissimilarities significantly increased with increased elevation. This suggests that the elevational α-diversity patterns for microbial taxonomic and functional genes are quite different, but that they may have similar elevational β-diversity patterns. While bacterial diversity/composition was strongly influenced by soil pH, microbial functional gene diversity/composition was significantly correlated with soil DOC. This finding highlights that soil DOC may be a good predictor of microbial functional gene elevational distribution. These results provide valuable information for our understanding of elevational diversity patterns and predicting the response of microbial functions to climate change. Coupling community and function is fundamental to advancing our understanding of ecology. We anticipate that our results will lead to more investigations of soil microbial communities from multiple viewpoints (taxonomic/phylogenetic/functional) to test existing or novel relationships and hypotheses.

## Author Contributions

HC conceived the idea. CS and YN conducted all the experiments for soil physicochemical analysis. CS collected soil samples. CS and YS conducted the data analysis. CS wrote the first draft and HC finalized the manuscript with assistance from all co-authors.

## Conflict of Interest Statement

The authors declare that the research was conducted in the absence of any commercial or financial relationships that could be construed as a potential conflict of interest.

## Footnotes

1 http://nbviewer.ipython.org/github/biocore/qiime/blob/1.9.1/examples/ipynb/illumina_overview_tutorial.ipynb

2 http://metagenomics.anl.gov/
