# Supplementary material for: Dramatic Increases of Soil Microbial Functional Gene Diversity at the Treeline Ecotone of Changbai Mountain
Source: Front Microbiol. 2016 Jul 29;7:1184. doi: 10.3389/fmicb.2016.01184 (PMC4965465; doi:10.3389/fmicb.2016.01184)

Table S1 Summary of the main climatic and ecological characteristics of the soil sampling sites along the elevational gradient on Changbai Mountain. MAT: mean annual temperature; MAP: mean annual precipitation.

| Forest type | Elevation (m) | Position | pH ranges | MAT (ºC) | MAP (mm) | Dominant tree species | Soil type |
| --- | --- | --- | --- | --- | --- | --- | --- |
|  |  |  |  |  |  |  |  |
| Broad-leaved forest | 500 | 42°42′38″N 128°14′07″E | 5.47-5.73 | 2.9 | 632 | *Quecus mongolica* | Albi-Boric Argosols |
|  |  |  |  |  |  |  |  |
| Mixed coniferous broad-leaved forest | 700 | 42°24′22″N 128°05′35″E | 5.07-5.8 | 2.6 | 691 | *Pinus koraiensis Acer tegmentosum* | Albi-Boric Argosols |
|  |  |  |  |  |  |  |  |
| Mixed coniferous broad-leaved forest | 1000 | 42°13′32″N 128°10′36″E | 5.81-6.31 | 1.8 | 759 | *Pinus koraiensis Acer tegmentosum* | Albi-Boric Argosols |
|  |  |  |  |  |  |  |  |
|  |  |  |  |  |  |  |  |
| Dark-coniferous spruce-fir forest | 1300 | 42°08′24″N 128°07′53″E | 4.84-5.19 | 0.3 | 811 | *Abies nephrolepis Picea jezoensis* | Bori-Udic Cambosols |
|  |  |  |  |  |  |  |  |
|  |  |  |  |  |  |  |  |
| Dark-coniferous spruce-fir forest | 1600 | 42°05′09″N 128°04′25″E | 4.58-5.06 | -2.3 | 967 | *Picea jezoensis* | Umbri-Gelic Cambosols |
| Ermans birch forest | 1900 | 42°03′31″N 128°03′57″E | 4.3-4.86 | -3.3 | 1038 | *Betula ermanii* | Permi-Gelic Cambosols |
|  |  |  |  |  |  |  |  |
| Alpine tundra | 2200 | 42°02′53″N 128°03′54″E | 5.26-5.49 | -4.8 | 1154 | *Dryas octopetala Rhododendron chrysanthum* | Permafrost cold Cambisols |
|  |  |  |  |  |  |  |  |

Table S2 Gene abundances of total and dominant functional gene categories in each sample.

| Sample | Total | C cycling | N cycling | Stress | Sulfur | Phosphorus | Virulence | Metal homeostasis | Organic remediation |
| --- | --- | --- | --- | --- | --- | --- | --- | --- | --- |
| 500-1 | 32904 | 5223 | 1367 | 5052 | 860 | 311 | 5358 | 8614 | 2947 |
| 500-2 | 33245 | 5289 | 1386 | 5108 | 869 | 324 | 5402 | 8674 | 2985 |
| 500-3 | 31281 | 4931 | 1312 | 4808 | 816 | 310 | 5127 | 8153 | 2818 |
| 500-4 | 32267 | 5077 | 1346 | 4948 | 846 | 312 | 5262 | 8438 | 2913 |
| 500-5 | 29737 | 4684 | 1237 | 4555 | 756 | 293 | 4946 | 7767 | 2661 |
| 700-1 | 30580 | 4846 | 1274 | 4686 | 799 | 299 | 5067 | 7972 | 2719 |
| 700-2 | 29847 | 4741 | 1255 | 4568 | 777 | 291 | 4928 | 7773 | 2672 |
| 700-3 | 31441 | 4982 | 1319 | 4799 | 816 | 305 | 5178 | 8200 | 2822 |
| 700-4 | 29079 | 4566 | 1216 | 4434 | 739 | 282 | 4831 | 7603 | 2608 |
| 700-5 | 30625 | 4846 | 1283 | 4684 | 804 | 298 | 5050 | 7992 | 2738 |
| 1000-1 | 32062 | 5079 | 1331 | 4889 | 842 | 313 | 5253 | 8370 | 2878 |
| 1000-2 | 31859 | 5051 | 1330 | 4877 | 836 | 312 | 5231 | 8319 | 2843 |
| 1000-3 | 30006 | 4734 | 1247 | 4597 | 782 | 296 | 4983 | 7831 | 2665 |
| 1000-4 | 31474 | 4986 | 1316 | 4826 | 829 | 311 | 5156 | 8214 | 2811 |
| 1000-5 | 29068 | 4578 | 1194 | 4447 | 749 | 285 | 4851 | 7586 | 2594 |
| 1300-1 | 30306 | 4764 | 1260 | 4658 | 784 | 294 | 5013 | 7912 | 2693 |
| 1300-2 | 30839 | 4855 | 1275 | 4734 | 801 | 299 | 5086 | 8074 | 2730 |
| 1300-3 | 31247 | 4942 | 1302 | 4797 | 810 | 308 | 5112 | 8164 | 2796 |
| 1300-4 | 31339 | 4978 | 1301 | 4818 | 818 | 295 | 5134 | 8194 | 2774 |
| 1300-5 | 29104 | 4610 | 1200 | 4464 | 747 | 282 | 4846 | 7605 | 2567 |
| 1600-1 | 31052 | 4933 | 1298 | 4734 | 812 | 294 | 5108 | 8076 | 2775 |
| 1600-2 | 28307 | 4465 | 1195 | 4348 | 726 | 269 | 4707 | 7350 | 2493 |
| 1600-3 | 31439 | 4978 | 1315 | 4816 | 820 | 299 | 5153 | 8217 | 2781 |
| 1600-4 | 27286 | 4312 | 1134 | 4188 | 699 | 260 | 4580 | 7098 | 2401 |
| 1600-5 | 33450 | 5324 | 1418 | 5109 | 880 | 315 | 5437 | 8731 | 2974 |
| 1900-1 | 49217 | 7905 | 2088 | 7611 | 1359 | 465 | 7574 | 12962 | 4344 |
| 1900-2 | 47932 | 7680 | 2032 | 7381 | 1319 | 451 | 7437 | 12617 | 4224 |
| 1900-3 | 46087 | 7381 | 1951 | 7097 | 1269 | 445 | 7130 | 12151 | 4093 |
| 1900-4 | 48990 | 7863 | 2083 | 7531 | 1357 | 470 | 7551 | 12908 | 4337 |
| 1900-5 | 49049 | 7860 | 2073 | 7582 | 1350 | 462 | 7568 | 12932 | 4343 |
| 2200-1 | 51771 | 8282 | 2210 | 7975 | 1448 | 489 | 7948 | 13703 | 4549 |
| 2200-2 | 49985 | 7988 | 2115 | 7720 | 1391 | 471 | 7693 | 13177 | 4435 |
| 2200-3 | 50872 | 8145 | 2177 | 7836 | 1420 | 483 | 7799 | 13453 | 4464 |
| 2200-4 | 50578 | 8083 | 2162 | 7750 | 1416 | 478 | 7786 | 13367 | 4475 |
| 2200-5 | 50779 | 8121 | 2172 | 7784 | 1416 | 479 | 7811 | 13421 | 4503 |

Table S3 Gene abundances of specific functional genesinvolved in carbon (C)-and nitrogen (N)-cycling in each sample.

| Sample | *nifH* | *nosZ* | *ureC* | *gdh* | Sylanase gene | *mcrA* | CODH gene | *tktA* | *pmoA* | Glucoamylase gene |
| --- | --- | --- | --- | --- | --- | --- | --- | --- | --- | --- |
| 500-1 | 194 | 133 | 165 | 54 | 136 | 31 | 94 | 179 | 15 | 57 |
| 500-2 | 196 | 140 | 164 | 58 | 141 | 30 | 95 | 185 | 15 | 56 |
| 500-3 | 180 | 133 | 158 | 57 | 129 | 27 | 91 | 173 | 14 | 54 |
| 500-4 | 192 | 135 | 162 | 57 | 135 | 31 | 94 | 176 | 15 | 59 |
| 500-5 | 173 | 124 | 147 | 52 | 119 | 26 | 91 | 169 | 14 | 48 |
| 700-1 | 180 | 126 | 155 | 56 | 128 | 24 | 86 | 168 | 15 | 54 |
| 700-2 | 173 | 123 | 150 | 53 | 124 | 25 | 84 | 162 | 14 | 51 |
| 700-3 | 188 | 129 | 157 | 57 | 131 | 26 | 90 | 171 | 14 | 56 |
| 700-4 | 167 | 118 | 144 | 52 | 125 | 26 | 85 | 159 | 14 | 45 |
| 700-5 | 181 | 124 | 155 | 55 | 126 | 23 | 89 | 167 | 14 | 55 |
| 1000-1 | 186 | 132 | 160 | 57 | 131 | 28 | 93 | 173 | 14 | 57 |
| 1000-2 | 185 | 136 | 160 | 57 | 132 | 29 | 92 | 177 | 13 | 53 |
| 1000-3 | 174 | 123 | 149 | 54 | 124 | 25 | 89 | 168 | 14 | 52 |
| 1000-4 | 185 | 133 | 153 | 57 | 129 | 26 | 93 | 177 | 14 | 55 |
| 1000-5 | 158 | 120 | 147 | 52 | 120 | 23 | 89 | 157 | 13 | 50 |
| 1300-1 | 174 | 125 | 146 | 52 | 120 | 23 | 86 | 166 | 14 | 56 |
| 1300-2 | 176 | 129 | 146 | 56 | 123 | 25 | 90 | 172 | 13 | 56 |
| 1300-3 | 176 | 127 | 151 | 54 | 124 | 28 | 92 | 172 | 13 | 58 |
| 1300-4 | 182 | 127 | 150 | 53 | 125 | 27 | 90 | 175 | 14 | 53 |
| 1300-5 | 163 | 105 | 145 | 52 | 119 | 26 | 86 | 160 | 14 | 55 |
| 1600-1 | 176 | 127 | 152 | 54 | 131 | 26 | 90 | 168 | 14 | 55 |
| 1600-2 | 163 | 117 | 143 | 52 | 117 | 23 | 85 | 158 | 13 | 46 |
| 1600-3 | 174 | 135 | 159 | 55 | 132 | 27 | 93 | 173 | 15 | 55 |
| 1600-4 | 146 | 108 | 141 | 49 | 114 | 25 | 85 | 150 | 14 | 48 |
| 1600-5 | 195 | 141 | 170 | 59 | 144 | 28 | 94 | 182 | 15 | 57 |
| 1900-1 | 308 | 215 | 238 | 83 | 212 | 45 | 129 | 262 | 27 | 79 |
| 1900-2 | 305 | 214 | 232 | 78 | 201 | 48 | 120 | 257 | 24 | 80 |
| 1900-3 | 283 | 198 | 224 | 78 | 189 | 45 | 117 | 250 | 23 | 79 |
| 1900-4 | 302 | 215 | 241 | 81 | 209 | 47 | 124 | 269 | 26 | 79 |
| 1900-5 | 298 | 216 | 237 | 82 | 209 | 43 | 122 | 266 | 25 | 83 |
| 2200-1 | 329 | 232 | 244 | 86 | 219 | 45 | 131 | 285 | 27 | 87 |
| 2200-2 | 316 | 223 | 232 | 79 | 213 | 46 | 123 | 272 | 26 | 85 |
| 2200-3 | 328 | 227 | 249 | 84 | 215 | 49 | 131 | 276 | 26 | 85 |
| 2200-4 | 320 | 229 | 241 | 86 | 213 | 48 | 126 | 280 | 28 | 83 |
| 2200-5 | 326 | 217 | 246 | 86 | 214 | 47 | 126 | 278 | 25 | 85 |

Table S4 Relative average abundances of bacterial phyla classified with RDP taxonomy across soils grouped into different elevations (values represents % of total sequences).

| **Taxon** | **500 m** | **700 m** | **1000 m** | **1300 m** | **1600 m** | **1900 m** | **2200 m** |
| --- | --- | --- | --- | --- | --- | --- | --- |
| k__Bacteria;p__AD3 | 0.22 | 0.27 | 0.39 | 0.22 | 0.74 | 0.52 | 0.23 |
| k__Bacteria;p__Acidobacteria | 16.45 | 15.99 | 18.05 | 28.45 | 25.01 | 24.51 | 17.13 |
| k__Bacteria;p__Actinobacteria | 5.54 | 5.40 | 7.17 | 4.87 | 4.17 | 4.82 | 9.49 |
| k__Bacteria;p__Armatimonadetes | 0.12 | 0.08 | 0.14 | 0.14 | 0.14 | 0.19 | 0.25 |
| k__Bacteria;p__BHI80-139 | 0.00 | 0.00 | 0.00 | 0.01 | 0.00 | 0.00 | 0.00 |
| k__Bacteria;p__BRC1 | 0.00 | 0.01 | 0.01 | 0.00 | 0.01 | 0.00 | 0.01 |
| k__Bacteria;p__Bacteroidetes | 5.58 | 4.43 | 6.91 | 7.04 | 3.34 | 8.39 | 4.62 |
| k__Bacteria;p__Chlamydiae | 0.20 | 0.28 | 0.92 | 1.04 | 0.46 | 0.54 | 0.49 |
| k__Bacteria;p__Chlorobi | 0.14 | 0.14 | 0.16 | 0.15 | 0.10 | 0.24 | 0.17 |
| k__Bacteria;p__Chloroflexi | 3.65 | 11.63 | 2.50 | 2.71 | 4.79 | 1.48 | 3.14 |
| k__Bacteria;p__Cyanobacteria | 0.09 | 0.09 | 0.12 | 0.21 | 0.10 | 0.10 | 0.12 |
| k__Bacteria;p__Deferribacteres | 0.00 | 0.00 | 0.00 | 0.00 | 0.00 | 0.00 | 0.00 |
| k__Bacteria;p__Elusimicrobia | 0.38 | 0.29 | 0.34 | 0.41 | 0.29 | 0.36 | 0.39 |
| k__Bacteria;p__FCPU426 | 0.01 | 0.01 | 0.01 | 0.07 | 0.05 | 0.04 | 0.01 |
| k__Bacteria;p__Fibrobacteres | 0.06 | 0.04 | 0.06 | 0.06 | 0.05 | 0.09 | 0.08 |
| k__Bacteria;p__Firmicutes | 6.97 | 7.05 | 6.95 | 7.32 | 6.16 | 8.15 | 7.90 |
| k__Bacteria;p__Fusobacteria | 0.00 | 0.00 | 0.00 | 0.00 | 0.00 | 0.00 | 0.00 |
| k__Bacteria;p__GAL15 | 0.00 | 0.01 | 0.00 | 0.00 | 0.00 | 0.00 | 0.00 |
| k__Bacteria;p__GN02 | 0.01 | 0.00 | 0.00 | 0.00 | 0.00 | 0.00 | 0.00 |
| k__Bacteria;p__GN04 | 0.00 | 0.00 | 0.00 | 0.00 | 0.00 | 0.00 | 0.00 |
| k__Bacteria;p__Gemmatimonadetes | 0.55 | 0.46 | 0.57 | 0.27 | 0.46 | 0.56 | 0.64 |
| k__Bacteria;p__Lentisphaerae | 0.02 | 0.02 | 0.01 | 0.01 | 0.01 | 0.02 | 0.01 |
| k__Bacteria;p__NC10 | 0.00 | 0.00 | 0.00 | 0.00 | 0.00 | 0.00 | 0.01 |
| k__Bacteria;p__Nitrospirae | 1.53 | 1.03 | 0.98 | 0.14 | 0.32 | 0.13 | 0.21 |
| k__Bacteria;p__OD1 | 0.39 | 0.23 | 0.68 | 0.33 | 0.10 | 0.10 | 0.49 |
| k__Bacteria;p__OP11 | 0.00 | 0.00 | 0.00 | 0.01 | 0.00 | 0.00 | 0.00 |
| k__Bacteria;p__OP3 | 0.09 | 0.12 | 0.20 | 0.05 | 0.05 | 0.03 | 0.08 |
| k__Bacteria;p__OP8 | 0.00 | 0.00 | 0.00 | 0.00 | 0.00 | 0.00 | 0.00 |
| k__Bacteria;p__Planctomycetes | 3.28 | 3.34 | 4.56 | 4.49 | 5.77 | 4.89 | 4.95 |
| k__Bacteria;p__Proteobacteria | 18.68 | 17.16 | 25.34 | 22.46 | 18.62 | 26.03 | 33.78 |
| k__Bacteria;p__SBR1093 | 0.00 | 0.01 | 0.00 | 0.00 | 0.00 | 0.00 | 0.00 |
| k__Bacteria;p__Spirochaetes | 0.24 | 0.22 | 0.18 | 0.16 | 0.19 | 0.43 | 0.27 |
| k__Bacteria;p__Synergistetes | 0.24 | 0.20 | 0.19 | 0.19 | 0.17 | 0.22 | 0.24 |
| k__Bacteria;p__TM6 | 0.16 | 0.08 | 0.28 | 0.45 | 0.23 | 0.21 | 0.12 |
| k__Bacteria;p__TM7 | 0.12 | 0.09 | 0.06 | 0.13 | 0.07 | 0.09 | 0.16 |
| k__Bacteria;p__Tenericutes | 0.09 | 0.10 | 0.11 | 0.09 | 0.08 | 0.19 | 0.15 |
| k__Bacteria;p__Thermotogae | 0.40 | 0.40 | 0.37 | 0.43 | 0.39 | 0.95 | 0.42 |
| k__Bacteria;p__Verrucomicrobia | 29.33 | 22.78 | 18.08 | 12.95 | 20.59 | 7.17 | 10.16 |
| k__Bacteria;p__WPS-2 | 0.06 | 0.11 | 0.12 | 0.22 | 0.40 | 0.28 | 0.20 |
| k__Bacteria;p__WS2 | 0.01 | 0.00 | 0.00 | 0.00 | 0.00 | 0.00 | 0.00 |
| k__Bacteria;p__WS3 | 0.18 | 0.11 | 0.10 | 0.03 | 0.05 | 0.01 | 0.02 |
| k__Bacteria;p__WWE1 | 0.29 | 0.23 | 0.35 | 0.29 | 0.25 | 1.22 | 0.37 |
| k__Bacteria;p__[Thermi] | 0.03 | 0.02 | 0.02 | 0.02 | 0.01 | 0.02 | 0.02 |

Table S5 Pearson correlations (R) between bacterial alpha diversity (OTUs, Faith’s PD) and microbial functional gene alpha diversity (functional gene richness, shannon index) and environmental variables. Values in bold indicate significant correlations (P < 0.05).TC: total carbon; TN: total nitrogen; DOC: dissolved organic carbon; DON:dissolved organic nitrogen; NH_4_^+^-N: ammonium; NO_3_^-^-N: nitrate; AP: available P; AK: available K.

| R | Elevation | Moisture | pH | AK | AP | NO3 | NH4 | DON | DOC | TN | TC |
| --- | --- | --- | --- | --- | --- | --- | --- | --- | --- | --- | --- |
| Bacterial  OTU richness | 0.07 | **-0.44** | **0.38** | -0.23 | -0.19 | **-0.4** | 0.05 | -0.15 | -0.26 | **-0.36** | **-0.36** |
| Faith's PD | -0.19 | 0.04 | **0.71** | **0.32** | 0.22 | -0.11 | 0.22 | 0.03 | 0.05 | 0.12 | -0.03 |
| Functional gene richness | 0.78 | **0.35** | -0.23 | 0.27 | **-0.49** | **-0.41** | -0.03 | **0.58** | **0.72** | 0.13 | **0.44** |
| Shannon index | 0.77 | 0.21 | -0.31 | 0.02 | **-0.53** | **-0.53** | -0.1 | **0.54** | **0.67** | -0.11 | 0.24 |

Table S6 Dissimilarities in bacterial community composition between elevations on Changbai Mountain as determined by analysis of similarities (ANOSIM) R values. An R value near +1 means that there is dissimilarity between the groups, while an R value near 0 indicates no significant dissimilarity between the groups. Values in bold indicate significant dissimilarity (P < 0.05).

| Elevation | 500 m | 700 m | 1000 m | 1300 m | 1600 m | 1900 m |
| --- | --- | --- | --- | --- | --- | --- |
| 700 m | **1.000** |  |  |  |  |  |
| 1000 m | **0.828** | **1.000** |  |  |  |  |
| 1300 m | **0.752** | **0.928** | **1.000** |  |  |  |
| 1600 m | **0.633** | **1.000** | **0.892** | **0.929** |  |  |
| 1900 m | **0.732** | **0.648** | **0.608** | **0.572** | **1.000** |  |
| 2200 m | **1.000** | **1.000** | **0.768** | **1.000** | **1.000** | **0.827** |

Fig. S1 A view of the treeline ecotoneon Changbai Mountain.


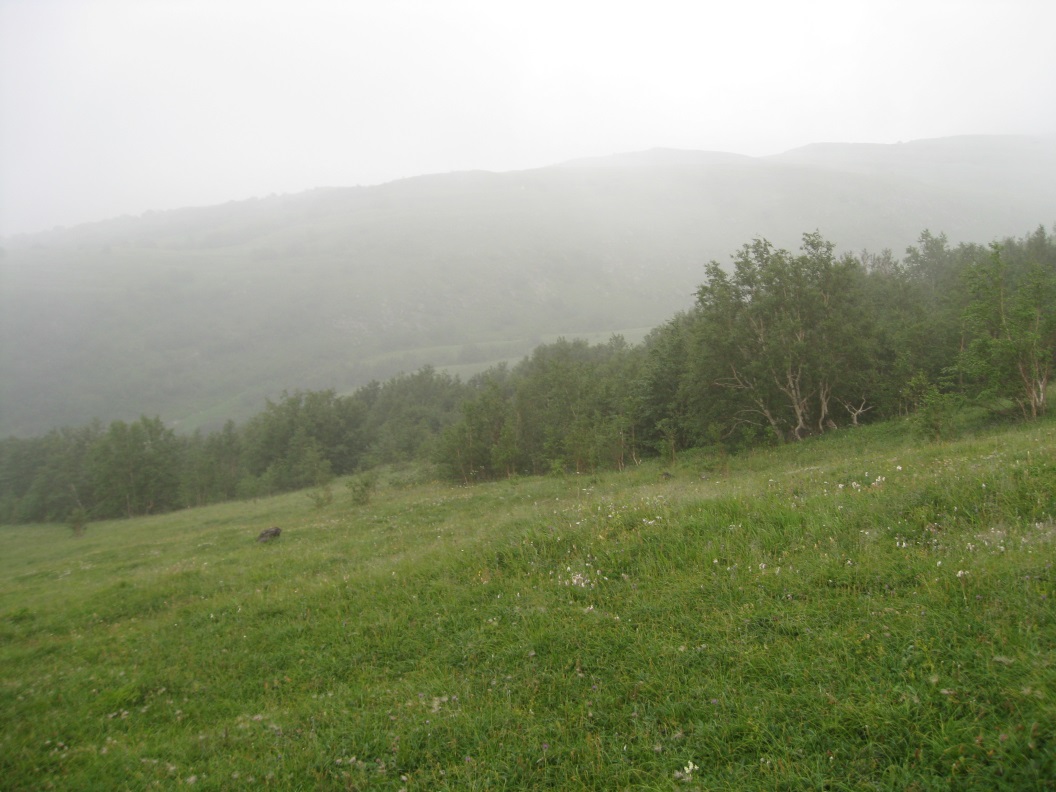


Fig.S2 Soil characteristics along seven elevation gradient on Changbai Mountain.


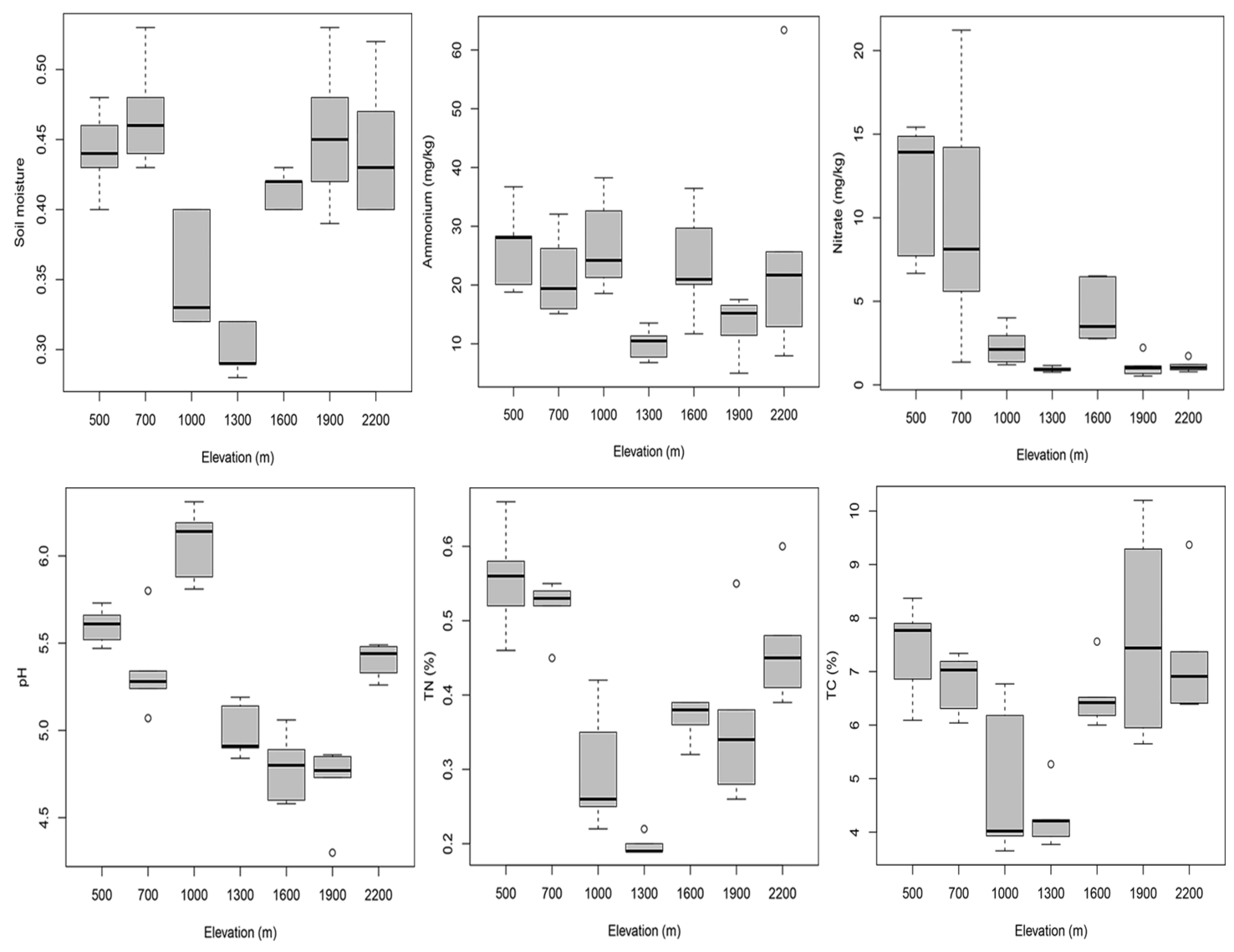


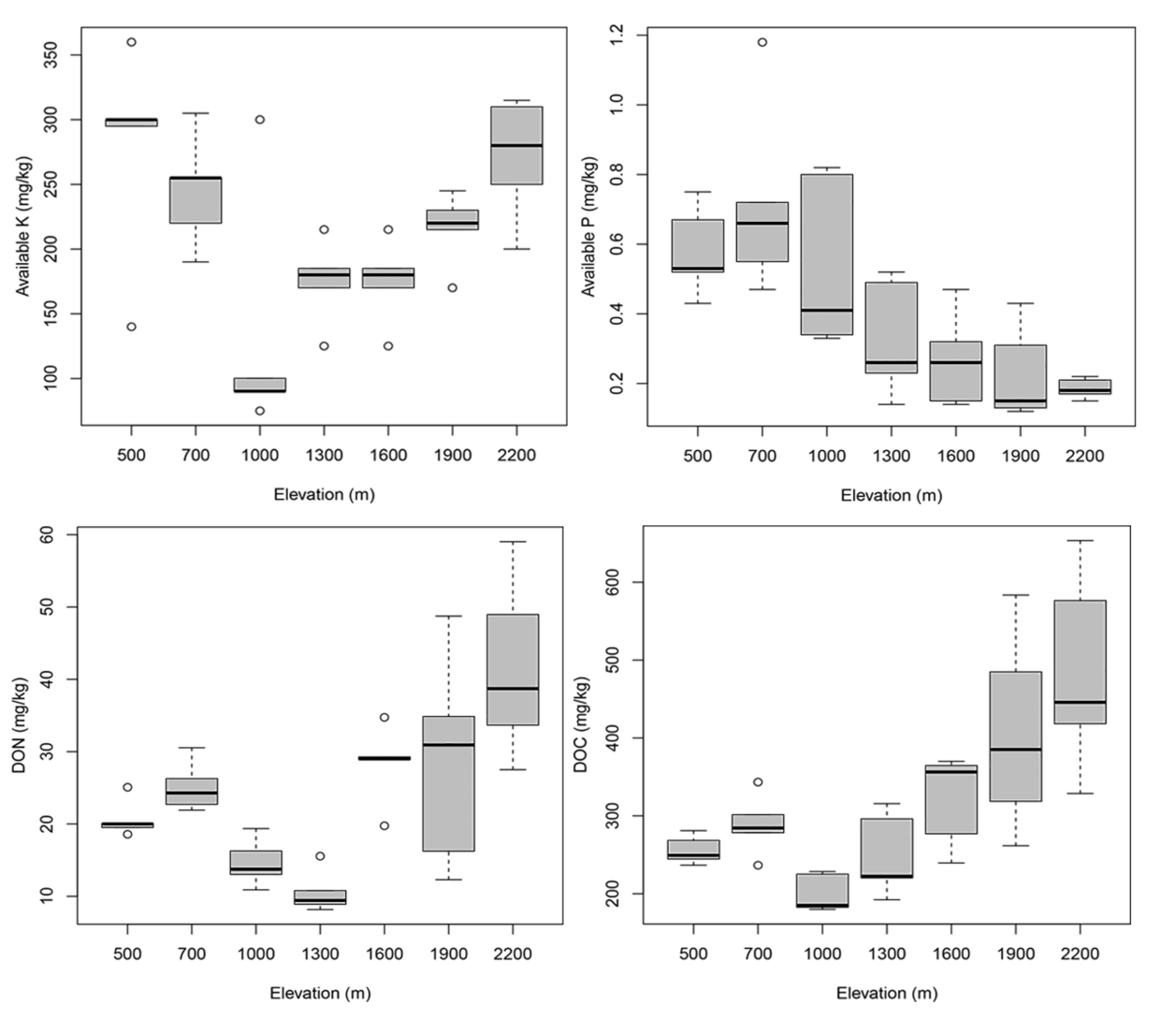


Fig. S3 Relative abundances of the dominant bacterial phyla in soils separated according to elevation categories. Relative abundances are based on the proportional frequencies of those DNA sequences that could be classified at the phylum level.


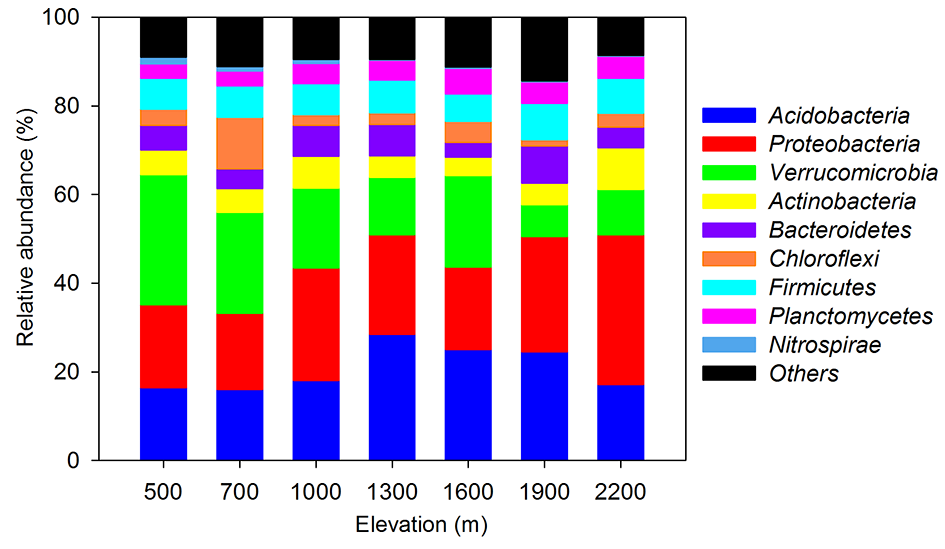


Fig. S4 The relationships between relative abundances of dominant bacterial groups and soil pH. Linear regressions were used to test the correlation between the taxa’s relative abundances and pH.


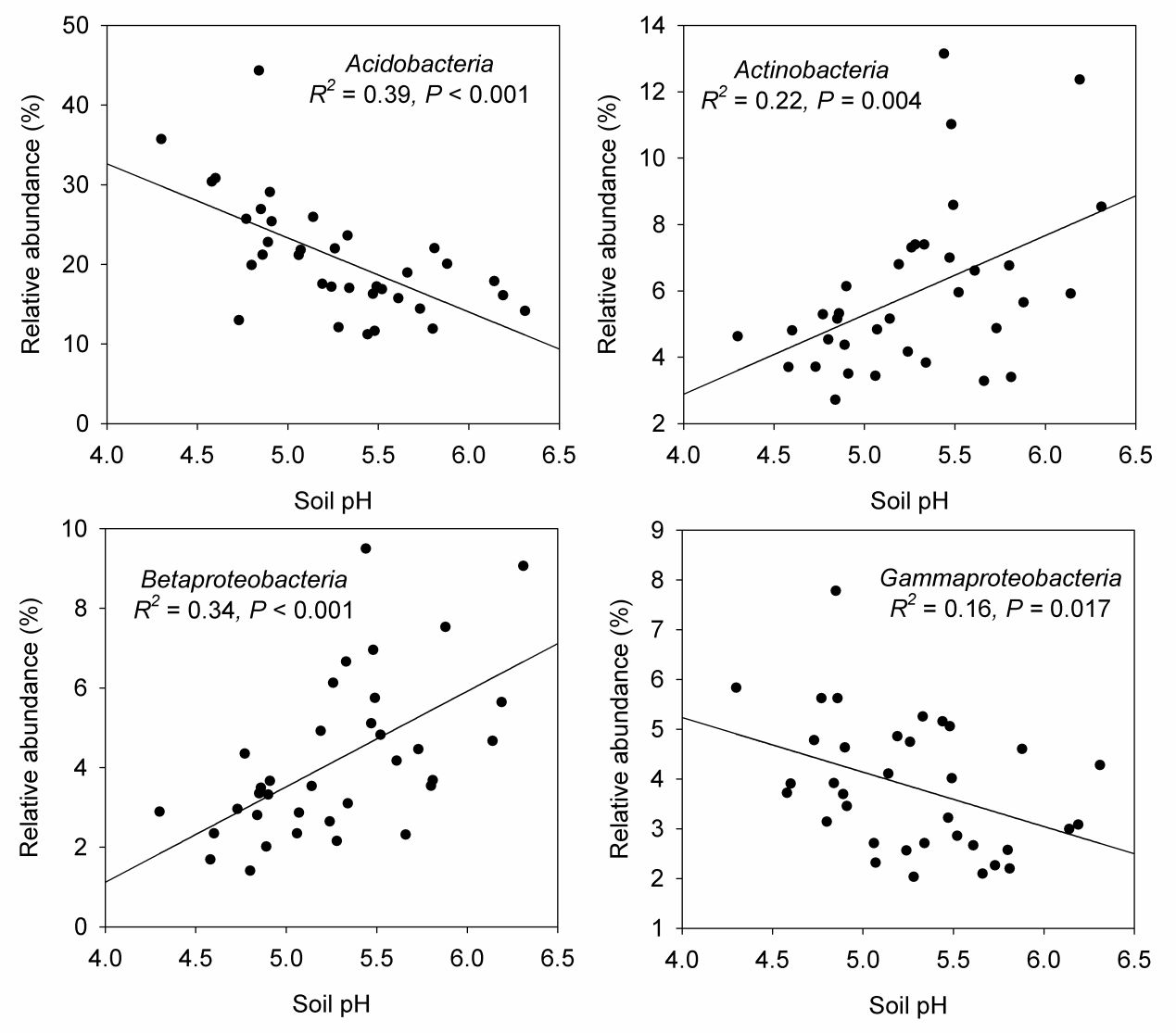

Supplement: Supplementary file 1 [file Data_Sheet_1.DOCX]
